# Hospital Saturday Fund

**Published:** 1891-12-26

**Authors:** 


### Hospital Saturday Fund.

The Board of Delegates of the Hospital Saturday Fund met at their central office, 59, Farringdon Road, Mr. R. B. D. Acland presiding, on Saturday night, when awards were made amounting in the aggregate to £16,000, which included £11,832 19s. granted to 27 general hospitals, £936 18s. to 32 dispensaries, and £1,418 14s. to 14 convalescent homes. A like total was distributed last year. Mr. Yerbury, in moving the adoption of the figures of the Distribution Committee, stated that the participating institutions had supplied the Committee with their latest yearly returns, which showed that the total number of new in-patients was 91,087; the average number of beds in use, 7,066; total number of new out-patients, 1,088,608; total number of attendances (inclusive of attendances of patients at home), 3,985,237. The largest awards were as follows:—

General Hospitals.—London, £829; Guy's, £551 11s.; St. George's, £458 10s.; St. Mary's, £367 5s.; Middlesex, £373 5s.; University College, £322 19s.; Westminster, £314 19s.; Seamen's, £295 14s.; King's College, £273 18s.; Royal Free, £252 18s.; Charing Cross, £233 9s.; West London, £189 17s.; German, £198; Great Northern Central, £129 19s.; Homœpathic, £113 5s.; North-West London, £139 16s.; Temperance, £132 19s.; West Ham, £111 12s.; Poplar (for accidents), £110 2s.; Metropolitan, £125 16s.; French, £88 1s.; Italian, £79 16s.; Miller Memorial, £86 1s.; and Deaconess's Institution (Tottenham), £100.

Special Hospitals.—*Cancer* (Brompton), £131 12s.; *Chest*, North London, £186; Brompton, £549 9s.; City of London (Victoria Park), £350 18s.; Infirmary for Consumption, £130 19s.; Royal for Diseases of the Chest (City Road), £220; Royal National (Ventnor), £108 13s.; National Sanatorium, £86 18s. *Children's.*—Alexandra, £135 2s.; Great Ormond Street, £214 18s.; East London, £156 6s.; Victoria, £150 8s.; Evelina, £112 3s.; Home and Infirmary for Sick Children (Lower Sydenham), £121 14s.; North-Eastern, £95 19s.; *Epilepsy*, &c.—National (Queen Square), £208 11s.; National for Diseases of the Heart and Paralysis, £75 15s. *Fistula*—St. Mark's, £79 14s.; Gordon, £53 17s. *Lock* Hospital, £150 19s. *Lying-in*—Queen Charlotte, £109 17s.; City of London, £104 3s.; and General, £87 17s. *Ophthalmic.*—Royal London, £205 18s.; a nd Royal Westminster, £103 5s. *Orthopœdic*—Royal, £109 16s.; and City, £80 10s. *Throat*—Central London, £84 17s. *Women*—Royal for Women, £120 17s.; and Samaritan Free, £101 10s.

Dispensaries.—Finsbury, £48 8s.; Islington, £48 15s.; Holloway, £44 3s.; Queen Adelaide, £43 5s.; Metropolitan, £39 7s.; Kilburn, £38 17s.; Chelsea, Brompton, and Belgrave, £36 17s.; and South Lambeth, £36 5s.

Convalescent Homes.—Morley House, £215 13s.; Metropolitan (Walton-on-Thames), £284 4s.; Bexhill, £141 3s.; Mrs. Gladstone's, £120; Clewer (St. Andrew's), £100; Folkestone (St. Andrew's), £110; St. Leonard's, £118 16s.; King's College, £64 3s.; and Princess Frederica's, £53 17s.

To the Surgical Aid Committee of the Hospital Saturday Fund £750 was awarded; to the Surgical Aid Society £115; and to the Provident Surgical Appliance Society, £95. The Distribution Committee have under consideration a scheme for sending children of subscribers to the Hospital Saturday Fund recovering from illness to convalescent and other homes upon a system of payment, the details of which plan will be laid before the Council at the first meeting in the coming year. The Lord Mayor (Alderman Evans) has consented to act as President of the fund during his term of office.

